# The intragenic mRNA-microRNA regulatory network during telogen-anagen hair follicle transition in the cashmere goat

**DOI:** 10.1038/s41598-018-31986-2

**Published:** 2018-09-21

**Authors:** Zhihong Liu, Feng Yang, Meng Zhao, Lina Ma, Haijun Li, Yuchun Xie, Rile Nai, Tianyu Che, Rui Su, Yanjun Zhang, Ruijun Wang, Zhiying Wang, Jinquan Li

**Affiliations:** 1 College of Animal Science, Inner Mongolia Agricultural University, Hohhot, 010018 China; 2 Key Laboratory of Animal Genetics, Breeding and Reproduction, Inner Mongolia Autonomous Region, Hohhot, China; 3 Key Laboratory of Mutton Sheep Genetics and Breeding, Ministry of Agriculture, Hohhot, China; 4 Engineering Research Center for Goat Genetics and Breeding, Inner Mongolia Autonomous Region, Hohhot, China; 5 College of Veterinary Medicine, Inner Mongolia Agricultural University, Hohhot, 010018 China

## Abstract

It is widely accepted that the periodic cycle of hair follicles is controlled by the biological clock, but the molecular regulatory mechanisms of the hair follicle cycle have not been thoroughly studied. The secondary hair follicle of the cashmere goat is characterized by seasonal periodic changes throughout life. In the hair follicle cycle, the initiation of hair follicles is of great significance for hair follicle regeneration. To provide a reference for hair follicle research, our study compared differences in mRNA expression and microRNA expression during the growth and repose stages of cashmere goat skin samples. Through microRNA and mRNA association analysis, we found microRNAs and target genes that play major regulatory roles in hair follicle initiation. We further constructed an mRNA-microRNA interaction network and found that hair follicle initiation and development were related to MiR-195 and the genes *CHP1*, *SMAD2*, *FZD6* and *SIAH1*.

## Introduction

Cashmere goats generate wool and cashmere, which are produced in the primary hair follicles and secondary follicles in the skin^1^. The growth of hair follicles is cyclical throughout the cashmere goat’s life^2^, undergoing cyclic transformations from stages consisting of anagen (rapid growth) to catagen (apoptosis-driven regression) and back to anagen^3^. Paus R proposed that a biological “clock” drives hair follicle cycling^4^. If so, what are the key players, and how many key players are molecular controls? The transformation and growth of cashmere are regulated by various complex factors in the skin^5–7^, and each follicle growth period in the skin has a specific activated/silenced gene expression pattern^8,9^.

MicroRNAs are a class of endogenous non-coding RNAs with lengths of approximately 20–24 nt^10^. These sequences combine with the sequences of mRNA-specific regions to complement target gene expression at the level of translation and play a role in the regulation of gene expression^11–13^. MicroRNAs play a role in regulating the cashmere growth cycle by targeting various signalling pathways and transcription factors^14^. MiR-203 regulates the differentiation of epithelial keratinocytes and directly inhibits the expression of p63^12,15^. MiR-200b and MiR-196a are associated with hair follicle development^16,17^. MiR-31 is associated with hair matrix differentiation and hair stem formation^18^, and its expression increases significantly during villus growth and decreases during degenerative and regenerative periods^19^. Hence, changes in microRNA expression patterns in skin cells are closely related to the cashmere growth cycle^20^.

The regulation of gene expression in life is relatively complex. Through analysis of microRNA sequencing and gene transcriptome sequencing data^21^, we can study the molecular mechanisms of microRNA-regulated gene expression systematically and obtain a more accurate understanding of the regulation of gene expression. In 2012, Liu performed microRNA sequencing of cashmere goat skin at various stages to determine whether microRNA expression varies in the skin or hair. Differences in the expression of microRNAs may occur through key gene regulation signalling pathways that modulate hair follicle growth^22^, and microRNA-mRNA interactions may regulate the development of hair follicles and their growth cycle^23^. In the present study, to clarify the regulatory mechanism of the cashmere growth cycle and gain insight into the related gene regulatory network, we characterized the interactions between microRNAs and mRNAs in goat skin, aiming to identify the key microRNAs regulating the growth and development of hair follicles and targeted mRNAs. We also characterized the gene regulatory network of differentially expressed microRNAs and their target genes, performed an annotated analysis of target genes related to differentially expressed genes, and explored key genes associated with hair follicle initiation and the regulation of microRNAs.

## Results

### Quality control of RNA and sequencing

**Figure 1:**
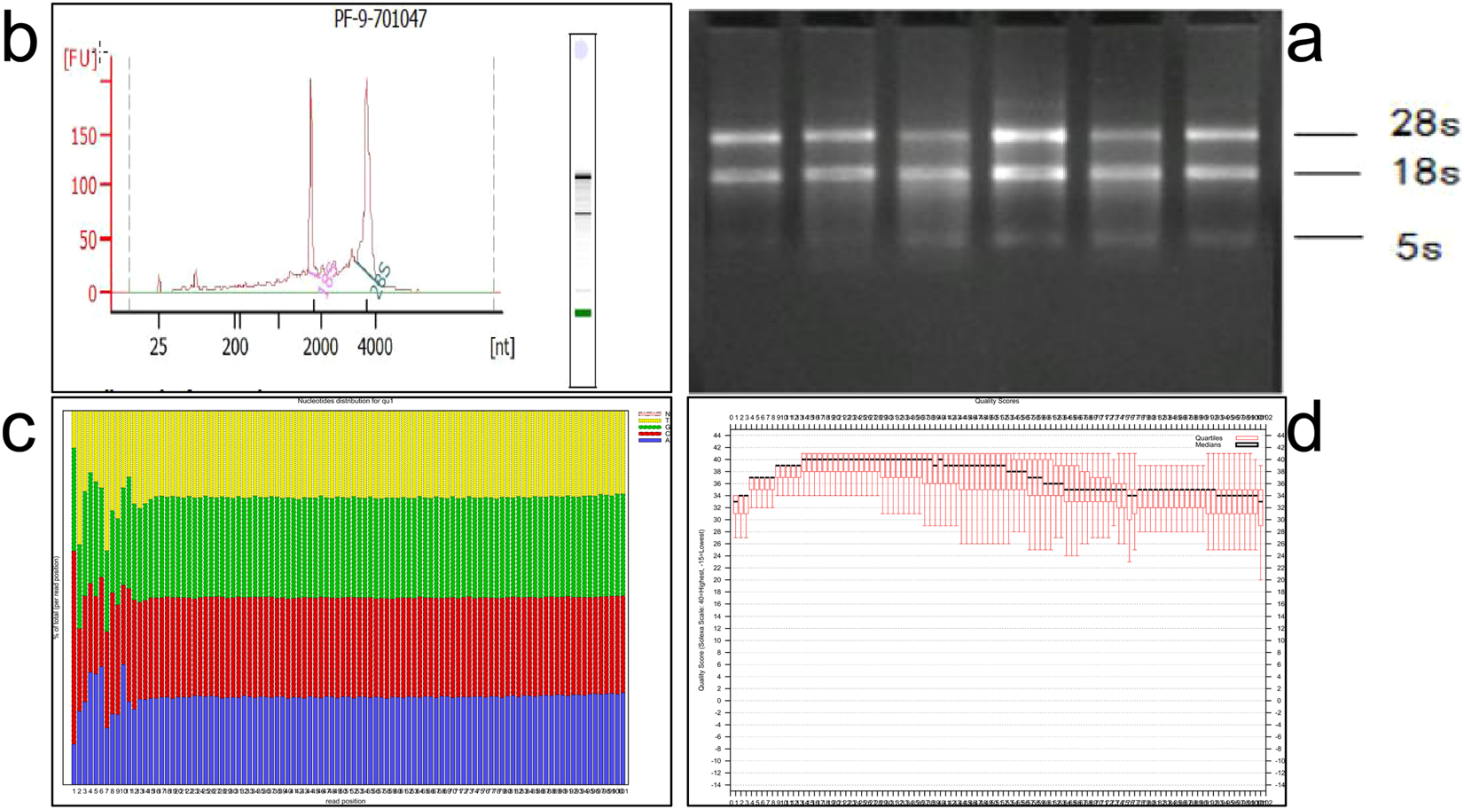
RNA quality inspection. (**a**) Agarose electrophoresis of total RNA from cashmere goat skin. (**b**) RNA electrophoretic and RIN values of the skin. (**c**) Base distribution of the original data; the abscissa is the reads base coordinate, and the ordinate is the percentage of A, T, C, G, and N bases among all reads. (**d**) Original data quality distribution; the abscissa coordinates are reads base coordinates, and the ordinate is the base mass of reads (Solexa scale: 40 = highest, −15 = lowest).

Total RNA samples from six Inner Mongolian Arbas cashmere goats (Fig. 1a) were analysed. Total RNA was not degraded, and there were no stray DNA bands. The OD260/OD280 ranged from 1.8 to 2.1, and the RNA integrity value exceeded 8.6. The RNA integrity met the sequencing requirements (Fig. 1b). The base distribution and mass fluctuation analysis of each circle sequence (Fig. 1c) showed that A, T, C, G, and N began to fluctuate at each location and then tended to stabilize. (The percentages of each base vary by species.) The base distribution of this study was uniform, the N% proportion was stable and low, and the base quality of the library was satisfactory (Fig. 1d).

### Transcriptome and MicroRNA data annotation

The amounts of transcriptome data in the telogen (T-1, T-2, and T-3) and anagen (A-1, A-2, and A-3) stages were 2.6 Gb, 2.3 Gb, 2.4 Gb, 2.4 Gb, 3.2 Gb and 2.4 Gb, respectively. After filtering out the low-quality sequences, we used Trinity software for de novo splicing to obtain the following amounts of transcriptome data: 198,542 bp, 219,454 bp, 219,411 bp, 247,695 bp, 250,081 bp, 247,695 bp and 203,451 bp. The total number of genes was 55,541, the number of alternative splices was 105,854, the average sequence length was 1965.15 bp, the length of the longest sequence was 23,311 bp, and the length of the shortest sequence was 351 bp.

The genes that control skin development differed significantly in the course of one year (Table 1). The first significant difference in gene number occurred from February to March, and the second occurred from March to April; thus, in March, there was a period of significant change for the genes in the skin, and these changes were relatively independent. The third significant change was from June to July, and the fourth significant change was October to November. After November and until February of the following year, changes in skin genes were almost absent.

**Table 1:** Statistical data for the differentially expressed genes between each pair of months.

	Jan.	Feb.	Mar.	Apr.	May.	Jun.	Jul.	Aug.	Sep.	Oct.	Nov.	Dec.
Jan.	0	19	3196	47	31	88	64	21	22	6	25	18
Feb.	19	0	1059	54	39	50	139	741	847	66	45	29
Mar.	3196	1059	0	731	2034	187	12865	8627	8820	5327	375	447
Apr.	47	54	731	0	13	17	68	1167	1234	228	100	84
May.	31	39	2034	13	0	1	9	102	141	50	105	74
Jun.	88	50	187	17	1	0	418	2844	2781	561	61	70
Jul.	64	139	12865	68	9	418	0	26	39	61	336	205
Aug.	21	741	8627	1167	102	2844	26	0	2	3	1469	897
Sep.	22	847	8820	1234	141	2781	39	2	0	1	1407	834
Oct.	6	66	5327	228	50	561	61	3	1	0	47	22
Nov.	25	45	375	100	105	61	336	1469	1407	47	0	8
Dec.	18	29	447	84	74	70	205	897	834	22	8	0

From sRNA sequencing of skin tissue samples from the repose and growth stages, we obtained telogen (T-1, T-2, T-3) and anagen data (A-1, A-2, A-3) consisting of 12.2 Mb, 11.0 Mb, 10.4 Mb, 11.3 Mb, 10.2 Mb and 10.9 Mb reads, respectively. After the removal of low-quality reads, we obtained 12.1 Mb, 10.6 Mb, 9.8 Mb, 11.2 Mb, 9.7 Mb and 10.8 Mb reads (Table 2) that could be used for subsequent analysis.

**Table 2:** Statistical results for sRNA sequencing data.

Class	T1		T2		T3	
	Quantity	%	Quantity	%	Quantity	%
Total Reads	12,269,110	100	11,034,263	100	10,433,558	100
Low Quality	30,829	0.25	33,284	0.3	42,878	0.41
adaptor3 null	28,448	0.23	110,453	1	139,022	1.33
insert null	46,229	0.38	132,781	1.2	214,081	2.05
5′ adaptor contaminants	1,077	0.01	2,468	0.02	8,713	0.08
size <18 nt	58,052	0.47	129,083	1.17	190,887	1.83
polyA	6	0	30	0	821	0.01
High Quality (size > = 18 nt)	12,104,469	98.66	10,626,164	96.3	9,837,156	94.28
**Class**	**A1**		**A2**		**A3**	
	**Quantity**	**%**	**Quantity**	**%**	**Quantity**	**%**
Total Reads	11,305,511	100	10,262,585	100	10,943,292	100
Low Quality	28,265	0.25	45,949	0.45	27,071	0.25
adaptor3 null	20,273	0.18	121,601	1.18	25,327	0.23
insert null	37,755	0.33	131,464	1.28	35,990	0.33
5′ adaptor contaminants	840	0.01	8,354	0.08	1,239	0.01
size <18 nt	60,332	0.53	190,460	1.86	59,557	0.54
polyA	18	0	549	0.01	24	0
High Quality(size > = 18 nt)	11,158,028	98.7	9,764,208	95.14	10,794,084	98.64

**Figure 2:**
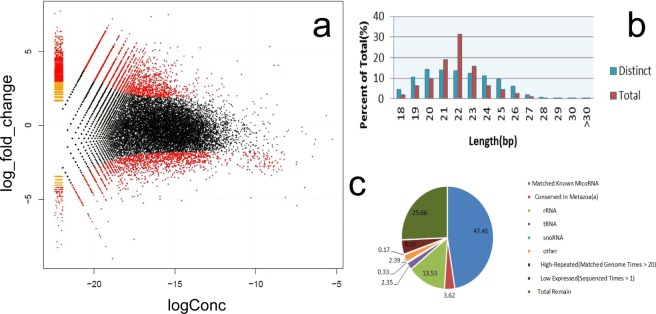
Sequencing data processing and analysis of differences. (**a**) Gene differential expression statistics; the ordinate is the Log_fold_change, the abscissa is the absolute expression level, that is, logConc. (**b**) SRNA high-quality reads length distribution statistics. (**c**) Comparison of sequence data with MiRbase database.

We also performed statistical analysis of the length distribution of the high-quality sRNA reads (Fig. 2b), and the results showed that total reads were primarily 22 nt long, accounting for 31.46% of reads, and that the proportions of reads that were 20, 21, 22, and 23 nt in length among distinct reads were 14.4%, 14.01%, 13.54% and 12.43%, respectively.

Comparing our high-quality sequencing data to known data in the MiRbase database (Fig. 2c), we obtained 437,517 known microRNA sequences, accounting for 47.41% of sequences, and 4,852,979 unknown microRNA sequences, accounting for 52.29% of sequences, which included rRNAs, tRNAs and snoRNAs, among others. There were 333 precursor microRNAs and 470 mature body microRNAs, which were classified into 150 families. We carried out a forecast of these new miRNAs and found that there were 92 precursor microRNAs and 99 mature body microRNAs.

### Differential analysis of the transcriptome and miRNAs

From a differential analysis of the differentially expressed genes that were related to villus growth in March and July (Table 1), we obtained 12,865 differentially expressed genes in the telogen and anagen stages, of which there were 7,664 upregulated and 5,201 downregulated genes (Fig. 2a). A differential analysis of mature microRNAs expressed during the growth and rest periods revealed 35 microRNA genes that were upregulated in the telogen stage compared to their expression in the anagen stage, and there were 16 genes with more than 100-fold higher expression in the anagen stage. We also found 9 downregulated microRNA genes in the anagen stage relative to the telogen stage, and there were 6 genes with more than 100-fold higher expression in the telogen stage: MiR-148a, MiR-34b, MiR-195, MiR-335, MiR-7g*-1, and MiR-101*-1. Thus, we successfully identified the major microRNAs and their target genes.

### Analysis of microRNA-mRNA interactions related to the initiation of cashmere production

Using transcriptome data as the target gene database, we obtained 12,927 differentially expressed microRNAs, among which there were 6,965 positive regulatory relationships and 5,114 negative regulatory relationships between the miRNA and the transcriptome. The minimum number of mRNAs with a target relationship with a single microRNA was 51, and the maximum number of mRNAs was 1682. The average number of target genes for a single microRNA was 783 (Table 3). Therefore, microRNA-mRNA interactions in cashmere goat skin were complex, and regulatory relationships can be expressed as one-to-one, one-to-many and many-to-many.

**Table 3:** MicroRNA and mRNA correlation analysis results.

Item	All (miRNA <−>mRNA)	Diff (miRNA <−> mRNA)	diff&negative(miRNA <−> mRNA)	Diff (miRNA <−> Pr)	diff&negative(miRNA <−> Pr)	Diff (miRNA < mRNA > Pr)	diff&negative (miRNA < mRNA > Pr)	Item	All (miRNA <−> mRNA)	Diff (miRNA <−> mRNA)	diff&negative(miRNA <−> mRNA)
Targets (Transcript)_Pr_edicted_by_MicroRNAs	12,927	6,965	5,114	117	92	72	39	Targets (Transcript)_Pr_edicted_by_MicroRNAs	12,927	6,965	5,114
Targets_number (min)_for_ONE_MicroRNA	51	24	4	1	1	1	1	Targets_number(min)_for_ONE_MicroRNA	51	24	4
Targets_number(max)_for_ONE_MicroRNA	1,682	997	559	17	10	10	6	Targets_number(max)_for_ONE_MicroRNA	1,682	997	559
Targets_number(average)_for_ONE_MicroRNA	782.73	424.45	175.79	6.89	3.4	3.28	2.07	Targets_number(average)_for_ONE_MicroRNA	782	424	175
MicroRNA_with_Predicted_Targets (Transcript)	56	56	56	54	52	39	29	MicroRNA_with_Predicted_Targets (Transcript)	56	56	56
MicroRNA_number (min)_for_ONE_Transcript	1	1	1	1	1	1	1	MicroRNA_number (min)_for_ONE_Transcript	1	1	1
MicroRNA_number (max)_for_ONE_Transcript	21	21	11	13	8	5	4	MicroRNA_number (max)_for_ONE_Transcript	21	21	11
MicroRNA_number (average)_for_ONE_Transcript	3.39	3.41	1.92	3.18	1.92	1.78	1.54	MicroRNA_number (average)_for_ONE_Transcript	3.39	3.41	1.92

### Mutual analysis of microRNAs and mRNAs

**Figure 3:**
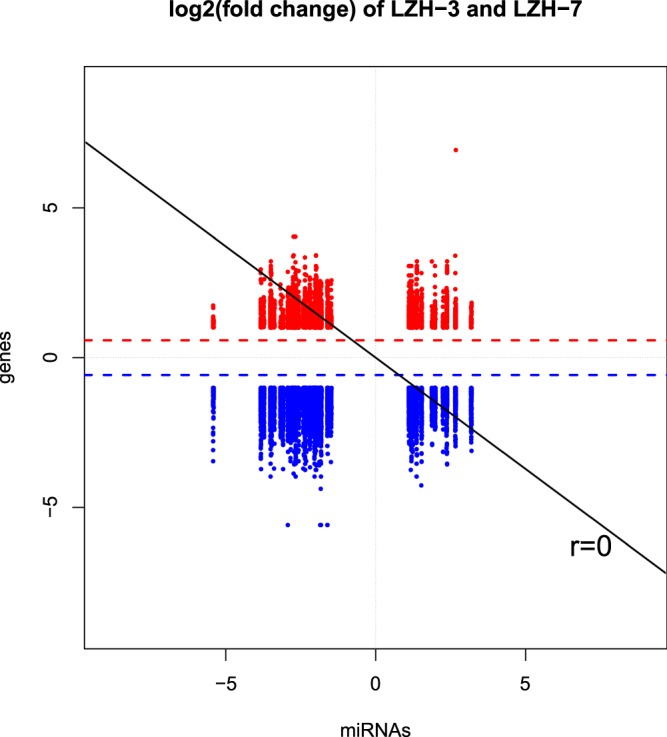
Correlation analysis of the positive and negative regulation of differential microRNA-mRNA expression. The abscissa is the log2 value of the multiple difference of target genes, and the longitudinal coordinate is the log2 value of the multiple difference of microRNAs. The red dashed line is 1.5 times the difference of the ascending line. The blue dashed line is 1.5 times the difference of the demarcation line.

The relationship between microRNA and mRNA is complex: one microRNA can target multiple mRNAs, and vice versa. A target gene can be regulated by multiple microRNAs simultaneously, and the relationship between microRNAs and mRNAs is not always limited to negative regulation. The mechanism by which microRNAs regulate mRNAs is not yet clear. This study explored the expression correlations between microRNAs and mRNAs through linear programming and determined the values of the differential multipliers for microRNAs and mRNAs (Fig. 3). For the first quadrant (upper right corner), there were 1,315 mRNAs and 43 microRNAs with higher expression in the anagen stage. For the second quadrant (upper left corner), there were 736 mRNAs with higher expression and 43 microRNAs with lower expression in the anagen stage. For the third quadrant (lower left corner), there were 449 mRNAs and 13 microRNAs with lower expression in the anagen stage. For the fourth quadrant (lower right corner), there were 890 mRNAs with lower expression and 13 microRNAs with higher expression in the anagen stage. Above all, the points in the second quadrant and the fourth quadrant were negatively associated with miRNA and mRNA. These miRNAs that were negatively related to mRNA will be our next area of study.

### Functional enrichment analysis of differentially expressed microRNA target genes

**Figure 4:**
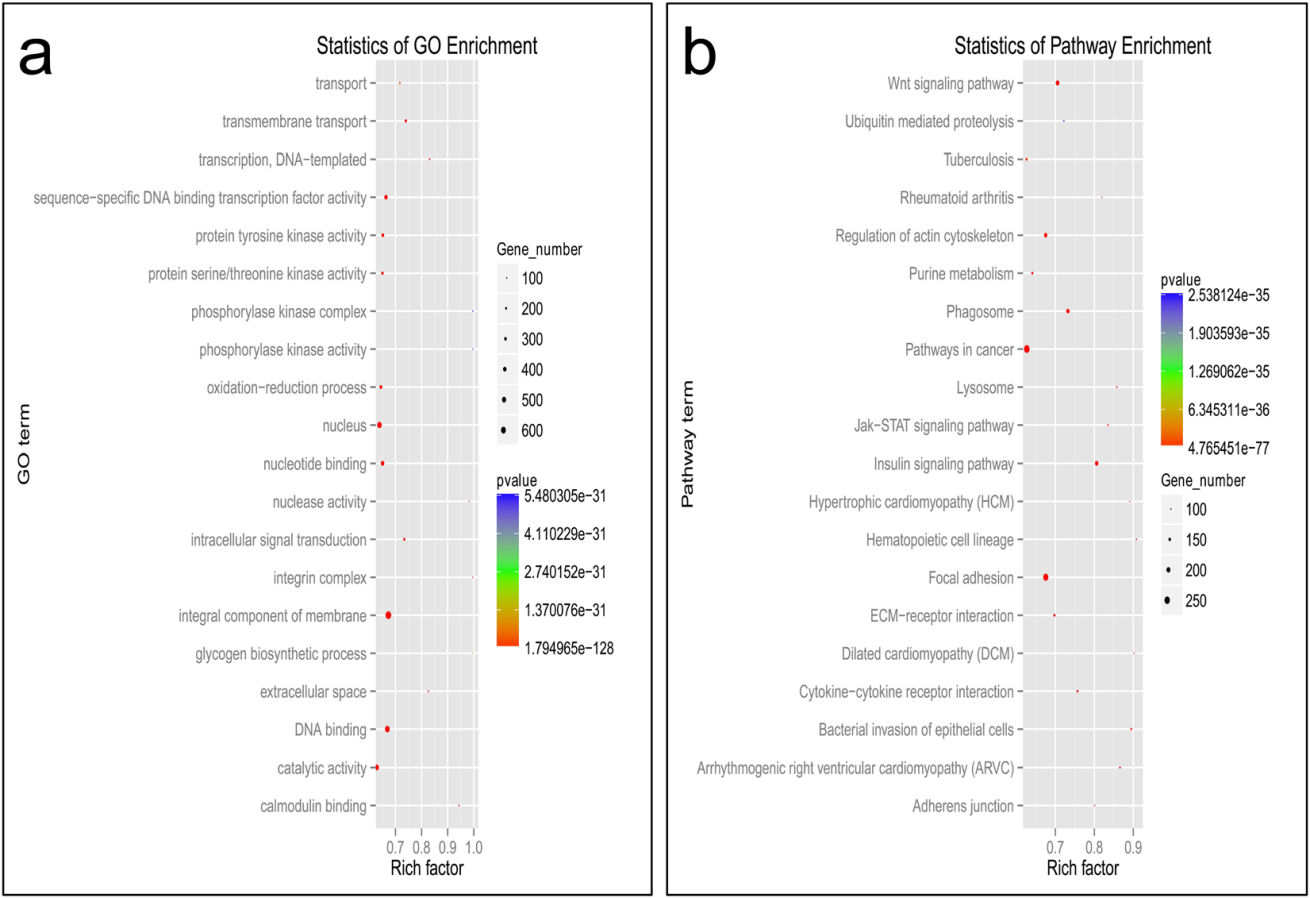
Enrichment analyses of GO orthology functions and KEGG pathways for differentially expressed microRNA target genes. (**a**) Function enrichment and analysis of target gene GO orthology for differentially expressed MicroRNAs. (**b**) Enrichment analysis of target gene KEGG pathways for differentially expressed MicroRNAs. The P value is the critical threshold for differential microRNA enrichment; the Rich factor is calculated for S/B, S is the mRNA with microRNA target relations annotated to a certain function or path, and B is used to annotate the mRNA of a function or path, based on the mRNA ratio. In other words, the Rich factor represents the ratio and is a percentage; the dot size represents the mRNA number, that is, S.

We also performed GO annotation and KEGG pathway analysis. As the results show, the GO database (Fig. 4a) included 8,409 differentially expressed mRNAs in the telogen relative to the anagen stage, of which there were 3,205 differentially expressed mRNAs upregulating microRNAs, and the number of differentially expressed mRNAs downregulating microRNAs was 4,723. The number of microRNAs negatively regulating their target mRNAs was 2,025. The KEGG database (Fig. 4b) included 5,432 differentially expressed mRNAs between the telogen and anagen stage; among these, there were 1,322 differentially expressed mRNAs upregulated by microRNAs.

**Figure 5:**
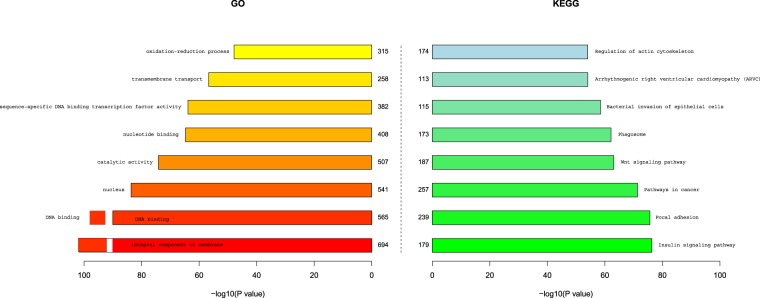
The first 8 results obtained for functional enrichment analysis of the differential expression of microRNA target genes. The length of the bar is the significantly enriched P value of the value (10 base); the smaller the P value, the more significant the enrichment, and the longer the length of the bar.

A collection of the most significant top 8 results for GO and KEGG enrichment is presented in Fig. 5.

The GO annotations of the most concentrated microRNA target genes were as follows, from high to low: integral component of the membrane, DNA binding, nucleus, catalytic activity, nucleotide binding, transcription factor activity, transmembrane transport and the oxidation-reduction process. These processes were related to cell development, indicating that a large number of cell proliferation processes occurred during the initiation of hair follicles.

The results of target gene KEGG annotation from high to low were as follows: insulin signalling pathway, focal adhesion, cancer pathways, Wnt signalling pathway, phagosome, bacterial invasion of epithelial cells, arrhythmogenic right ventricular cardiomyopathy and regulation of the actin cytoskeleton. Similar to the results obtained with KEGG pathway enrichment, we found that the concentration of insulin signalling pathways was the highest; there were 220 genes annotated to this pathway. Although there have been no reports of the role of insulin signalling in hair follicle development to date, the insulin signalling pathway is closely related to cell growth, and its potential involvement in hair follicle development will be further studied. We also analysed the signalling pathways related to hair follicles, including the Jak-STAT signalling pathway, Notch signalling pathway and Wnt signalling pathway, which were previously reported. The corresponding numbers of genes identified by annotation were 123, 348 and 262, respectively. The function enrichment P value of the Wnt signalling pathway was 7.29E-64, which indicated a closer relationship with villus development, as shown by the large number of genes in the signalling pathway in skin tissue exhibiting targeted regulatory relationships with the differentially expressed microRNAs. Therefore, Wnt signalling plays an important role in the development of hair follicles.

### mRNA-microRNA network analysis

**Figure 6:**
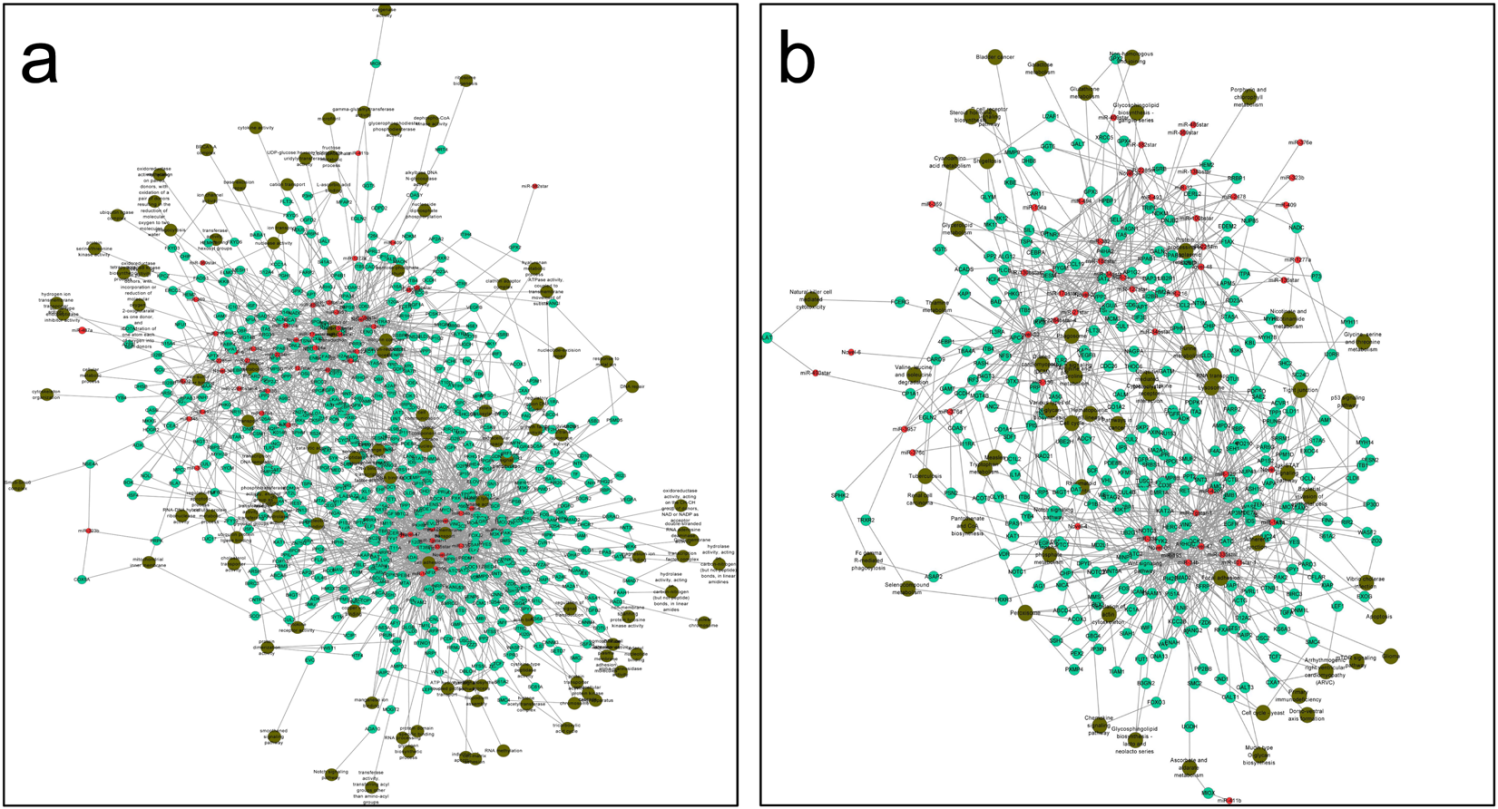
Network analysis of mRNA-microRNA regulation. (**a**) Analysis results for the microRNA-mRNA enriched GO function network. (**b**) MicroRNA-mRNA KEGG pathway enrichment network. The dark dots are signalling pathways, the small green dots are differentially expressed genes, and the red dots are microRNAs with target regulation relationships.

The telogen and anagen stages in cashmere goat skin showed differential expression of microRNAs and their negatively regulated target genes according to the construction of a data relation network based on GO annotation and network construction based on KEGG relationships (Fig. 6). Genes differentially expressed in the telogen and anagen stages have many functions in cashmere goats (Fig. 6a), comprising a complex regulatory network with microRNAs. With GO annotation, there were 350 upregulated genes and 13 microRNAs clustered in the lower part of the network. Additionally, 233 downregulated genes and 33 microRNAs were clustered above the network. Among GO network relationships, genes related to cashmere growth in the Wnt signalling pathway were *FZD6*, LEF1, FZD3, WNT5A and TCF7, and related microRNAs were MiR-195, MiR-148a, MiR-4206 and gamma.

The KEGG pathway network diagram was similar to the GO annotation network diagram (Fig. 6b). The genes were clearly divided into two groups, with 184 upregulated genes and 13 microRNAs clustered in the lower part of the network. Fourteen genes were annotated in the Wnt signalling pathway, including calcineurin B homologous protein 1 (*CHP1*), E3 ubiquitin-protein ligase (*SIAH1*), *FZD6*, WNT5A and mothers against decapentaplegic homolog 2 (*SMAD2*), as well as MiR-195, MiR-335star and other microRNA genes. One hundred twenty-eight downregulated genes and 31 microRNAs were clustered above the network. The genes and microRNAs annotated in the Wnt signalling pathway were 2A5G, CUL1, LRP5, FOSL1, MiR-17astar, MiR-2285m, MiR-136 and MiR-34astar. Therefore, there is a complex signalling pathway between the genes expressed during rest and the genes downregulated during the growth stage.

Up- and downregulated target genes as well as the negative regulatory effects of microRNAs during the period of transition from the telogen to the anagen stage were clearly clustered into two groups. The brown dots in the network map indicate the functional annotation of target genes. Some functions in the map were annotated only for upregulated genes, some functions were annotated only for downregulated genes, and others had functions in both up- and downregulated transcription. This finding indicates that the thresholds for gene balance or gene expression and for differentially expressed genes were relatively important for a specific function. The interactions between microRNAs and their target genes played roles in the initiation and growth of cashmere. This finding further indicates that the initiation of hair follicle growth was controlled by multiple genes and that microRNAs play a role in the post-transcriptional negative regulation of target genes in hair follicle initiation.

### Construction of a control network for the Wnt signalling pathway

**Figure 7:**
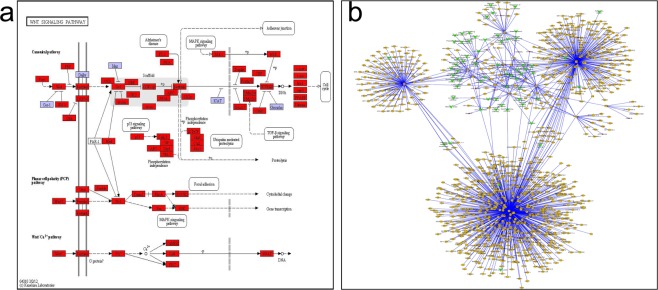
Wnt signal path annotation results and construction of the regulatory network. (**a**) Wnt signal pathway^51–53^ (http://www.kegg.jp/kegg/kegg1.html.). Rectangular nodes represent gene products (such as enzymes or some RNA regulator), gene products all belong to the KEGG ONTOLOGY classification system indicated with a blue background (KO) (some have highly similar sequences, and the same pathway proteins with similar functions were classified as a group KO), and gene products on the white background are not part of the KO classification system. Sequencing of the genes annotated in red revealed these non-KO gene products (nodes indicate gene products with the same or similar functions); circular nodes represent compounds (i.e., a substrate or product); a white background with a rounded rectangle indicates another pathway. The arrow shows the direction of the enzyme reaction or the direction of information transfer, the solid line indicates a direct solution, and the dotted line indicates an indirect solution. (**b**) Wnt signalling pathway regulation network.

Multiple genes and microRNAs were involved in the Wnt signalling pathway during goat hair follicle development (Fig. 7a). In addition, the genes and microRNAs in the Wnt signalling pathway also played roles in other related pathways and interacted with other genes. In this study, we used a “biogrid” database to download the genes that interacted with target genes and then merged the genes from the Wnt signalling pathway to construct the Wnt signalling network (Fig. 7b).

Using the GO regulatory network, KEGG regulatory network and the Wnt signalling pathway regulatory gene interaction network in the goat hair follicle resting phase, we screened for genes with upregulated expression in the Wnt signalling pathway from the telogen to the anagen stage, including *CHP1*, *FZD6*, *SIAH1* and other genes. Keratin and keratin-associated proteins were constitutive proteins during cashmere production. MiR-195 was predicted using target gene prediction and found to negatively regulate KAP24-1, KAP3-1, KAT8 and the important regulatory factor EGFR, which constitute the main components of cashmere. The genes corresponding to KAP24-1, KAP3-1, KAT8 and EGFR were *CHP1*, *FZD6*, *SIAH1* and *SMAD2*. Therefore, we chose *CHP1*, *FZD6*, *SIAH1* and *FZD6* as the regulatory genes during the initiation of villus growth for further verification.

### Expression of *CHP1*, *FZD6*, *SIAH1*, and *SMAD2*

**Figure 8:**
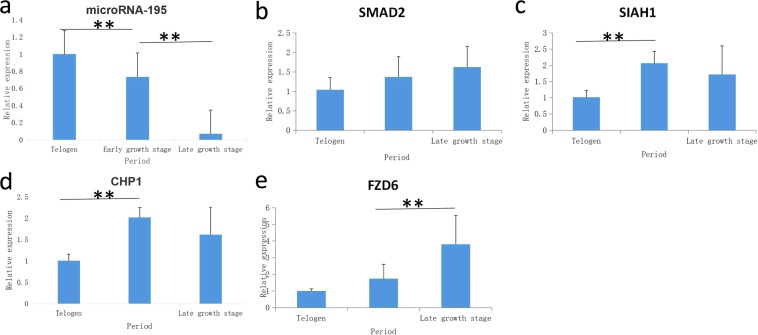
Fluorescence quantitative expression results. **Indicates the difference between representatives is very significant, and * indicates the difference is significant. (**a**) Relative expression of microRNA-195. (**b**) Relative expression of *SMAD2*. (**c**) Relative expression of *SIAH1*. (**d**) Relative expression of *CHP1*. (**e**) Relative expression of *FZD6*.

The expression of *CHP1*, *FZD6*, SIAH, and *SMAD2* was verified via fluorescence quantitative PCR, using β-actin as a reference gene. As shown in Fig. 8, compared to the expression of microRNA-195 (Fig. 8a), the expression level of the *CHP1* gene (Fig. 8d) was higher in the anagen stage than in the telogen stage, and the difference was relatively significant (P < 0.01), with a descending trend from the early growth stage to the late growth stage that was not significant (P > 0.05). The expression level of the Frizzled-6 (*FZD6*) gene (Fig. 8e) increased from the early stage to the telogen stage, but the difference was not significant (P > 0.05). However, the expression level increased significantly from the early growth stage to the late growth stage (P < 0.01). The expression of the gene *SIAH1* (Fig. 8c) increased significantly from the telogen to the early growth stage (P < 0.01), but there was no significant difference from the early growth stage to the late growth stage (P > 0.05). The *SMAD2* gene (Fig. 8b) showed no significant difference from the telogen to the anagen stage (P > 0.05), but its expression increased from the telogen to the late growth stage. In the Wnt signalling network, two of the three key genes were significantly expressed from the telogen to the anagen stage.

## Discussion

Farazi T A *et al*. showed that a computer-aided algorithm could be used to integrate miRNA and mRNA paired expression spectra to characterize miRNA-mRNA interactions and that this approach identified miRNA targets with high accuracy. This approach effectively avoids certain software predictions that do not consider specific cases of miRNA and mRNA expression^24–26^. Chen *et al*. published an article describing a model for predicting disease associations by mixing microRNAs into computations (HAMDA), which significantly reduced experimental time and cost^27–29^. With HAMDA, the functions of microRNAs can be correlated with disease characteristics, and the role of new microRNAs in disease can be revealed more clearly. Additionally, the authors proposed a method of using Laplacian Regularized Sparse Subspace Learning for MicroRNA-Disease Association (LRSSLMDA), which projects a microRNA and a disease into a common subspace to interpret the function of the microRNA subspace^27,30–32^. Based on these methods and on a joint analysis of miRNA-mRNA, we conducted a targeted prediction of miRNA-mRNA interactions and used reverse locations of mRNAs to predict miRNA target genes. After obtaining positive target genes, we used RT-qPCR to further verify the target genes and determine the interactions between target genes regulated by miRNAs.

In this study, target genes of differentially expressed microRNAs were analysed using GO orthology enrichment analysis and KEGG pathway enrichment^33^, as described in Chen L’s study. The most commonly annotated GO function was integral component of the membrane within cellular components, indicating that microRNAs can regulate villus initiation and the villus growth cycle by regulating cell membrane-associated gene expression. The KEGG pathway results showed significant enrichment of the Jak-STAT signalling pathway, Notch signalling pathway, p53 signalling pathway and Wnt signalling pathway. These cashmere growth-related pathways were found via analysis of differentially expressed microRNA target genes in the KEGG pathway map, and the Wnt signalling pathway was found to play a particularly important role. Therefore, this study constructed the Wnt signalling pathway regulatory network during hair follicle initiation.

The American sociologist Granovetter first posited the concept of relationship intensity and divided relationships into strong and weak relationships^34^, with strong ties maintaining the relationships between groups and organizations and weak ties between groups and organizations. Information obtained from strong relationships is often highly repeatable, while weak relationships provide more information and other resources than strong relationships. Based on the ideas provided by social networks, we hypothesize that strong and weak relationships exist between genes. A gene associated with more genes in a network is probably an important gene or key node gene that controls or influences a trait^35^. Thus far, efforts such as pathway analysis have raised awareness of the functional contributions of gene mutations and DNA copy number variations to cancer development, progression and metastasis^36^. Wang E commented on the challenges associated with studying cancer omic data using an integrative network approach and suggested possible research directions. As a result, most current cancer genome sequencing work has mapped mutations onto biological pathways, including signalling pathways^37,38^. Mcgee S R indicated that by studying the distributions of such network motifs, insight into cancer signalling regulatory molecular mechanisms of tumorigenesis can be gained, and the identification of these loops has practical implications for predicting prognosis and the clinical outcomes of cancer patients. Collectively, network motifs and modules are critical for cancer signalling and are associated with clinical outcomes^39^. Additionally, as stated by Han P, analysis of GRNs can identify regional subnetworks for certain biological processes; in-cloud regulatory structures between genes, key regulators, and cancer hallmark subnetworks; network dynamics for network rewiring; and network motifs. Furthermore, such results may reveal the molecular mechanisms of signalling pathways associated with cancer hallmarks and cancer patient outcomes^38,40–42^. We found a complex network between genes in the Wnt signalling pathway and other genes and signalling pathways during hair follicle development from rest to growth, indicating that hair follicle initiation is determined not by single genes but by differences in the number of interactions between genes. One or more key genes are present in this gene regulatory network. In the Wnt signalling pathway, gene interactions are centred on the *CHP1*, *SIAH1*, and *SMAD2* genes; however, many inter-gene interactions and multiple signalling pathways are involved.

As indicated by previous studies, microRNAs have negative regulatory roles^43–46^. In this study, quantitative analysis of MiR-195 expression during each period of chorionic villus initiation was performed. MiR-195 showed a trend of declining expression during the periods of repose, prophase and growth. In our experimental results, we found that two genes, *SMAD2* and *FZD6*, were negatively correlated during the periods of repose, prophase and growth. Therefore, we speculate that MiR-195 continues to inhibit the expression of *SMAD2* and *FZD6* during the entire initiation process. At present, no information is available regarding the regulation of *SMAD2* and *FZD6* targeting in by-195. However, a study by Mo J showed that MiR-195 regulates cell proliferation (Mo J. *et al*.^47^). Zheng R., Du J. *et al*. showed that downregulated MiR-195 was targeted to promote the expression of SMAD7 in two-leaf aortic valve calcification^48,49^. MiR-195 may play an important role in the regulation of SMAD family genes. However, two genes, *SIAH1* and *CHP1*, showed a trend that first increased and then decreased. Presumably, MiR-195-mediated inhibition of these two genes after the initiation of cashmere production is affected by other microRNAs and is relieved or reduced. A study by Zhang X showed that MiR-195 affects the proliferation of colon cancer cells and regulation of Wnt/β-catenin pathway protein-specific MiR-195 by targeting FGF2 and regulation of the Wnt/β-catenin signalling pathway, consistent with studies showing the effects of MicroRNA-195 on Wnt signalling^47,50^.

## Materials and Methods

### Animals

The study samples were collected from an Inner Mongolian Arbas white cashmere goat breeding farm. All animal experiments were performed in accordance with the ‘Guidelines for Experimental Animals’ of the Ministry of Science and Technology (Beijing, China). All surgeries were performed according to recommendations proposed by the European Commission (1997) and were approved by the experimental animal ethics committee of Inner Mongolia Agricultural University. Six cashmere goats were selected from the same growth environment and had equal body weights, unrelated relationships, were of the same sex, and had good growth conditions. The sampling times occurred during the telogen and anagen stages. The sampling position was the side body or the middle of the body at a distance of 10–15 cm from the scapula, and the sample size was 2 cm^2^. The samples were stored at −80 °C in a freezer.

## Methods

### Sequencing

Extraction of total RNA with liquid nitrogen lapping and RNAiso Plus (Takara, China), with separation on a Bioanalyzer 2100 (Agilent, USA), was used to detect RNA and microRNA quality. A cDNA library was constructed for the transcriptional group using a TruSeq^TM^ RNA sample preparation kit (Illumina, USA) according to the manufacturer’s instructions. A microRNA sequencing library was constructed according to the instructions of the Illumina (USA) reference kit (USA). The transcriptome was constructed, and Solexa sequencing of the microRNA library was performed by Shanghai M-image Biological Medicine Science and Technology Co., Ltd.

### Original data processing and differential expression analysis

Seqprep, sickle, and condetri_v2.0.pl software were used to evaluate the original data for transcriptional sequencing, and Trinity software was used for de novo splicing and open reading frame (ORF) prediction. We predicted ORF sequences using BLAST (BLAST Version 2.2.25, with an E-value parameter value setting of less than 10^−5^) in the NCBI NR database with BLASTP database identification of the string database and KEGG database without predicted sequences or NR, string, or gene library ORF BLASTX comparison notes. The expression of each gene in each sample was counted based on a comparison between the sequencing sample and the reference genome, and gene expression levels were calculated using the maximum likelihood method in RSEM software. EdgeR software was used to find significant differences in the expression of all transcripts in the sample at a threshold of P = 0.05.

Short oligonucleotide alignment program (SOAP) software was used to locate sRNAs in the genome. “Tag2repeat” software was used for repeated sRNA comparisons. Using overlap software, sRNAs were compared to the exons and introns of mRNAs, and sRNAs derived from mRNA degradation were found. The GenBank and Rfam (9.1) databases (database link) were used to annotate the data and remove rRNAs, scRNAs, snoRNAs, snRNAs, and tRNAs from the sRNAs as much as possible. The sequences were compared to microRNA precursors and the mature bodies of cattle and sheep in MiRBase21 (http://www.MiRbase.org/) to obtain known microRNAs. We analysed whether there were significant differences in the expression of known microRNAs (P = 0.05) and compared microRNA expression patterns using a log 2-ratio and scatter plot.

### Association analysis

#### Target gene prediction for the initiation of related microRNAs

Targetscan (parameter: context score percentile of ≥90) and MiRanda (parameter: max energy of ≤−20) software were used to predict the differentially expressed microRNA target genes in the telogen and anagen stages, and the target regulatory relationships between microRNAs and mRNAs were determined based on sequence complementarity, sequence conservatism, thermo-kinetic factors, site-binding ability and UTR base distribution. The relationships between microRNAs and mRNAs are complex. A microRNA can target multiple mRNAs simultaneously. In contrast, a target gene mRNA can be regulated by multiple microRNAs simultaneously. We used ACGT101-CORR (1.1 version) software to extract and perform statistical analysis for data describing target regulation relationships, specifically positive and negative relationships between the expression of microRNAs and their target genes expressed as the difference multiplier of microRNA and the difference multiplier of mRNA (R correlation analysis).

#### Analysis of genes related to chorionic cycle initiation

This study used GO enrichment and KEGG functions for gene analysis. The target functions of all genes or annotations among the differentially expressed miRNAs were counted, and hypergeometric tests were used to determine the functions or pathways that were significantly enriched among differentially expressed miRNA-mRNA relationships. The formula for calculating P value significance was

$${\rm{P}}=1-{\sum }_{{\rm{i}}=0}^{{\rm{S}}-1}\frac{(\frac{{\rm{B}}}{{\rm{i}}})(\frac{{\rm{TB}}-{\rm{B}}}{{\rm{TS}}-{\rm{i}}})}{(\frac{{\rm{TB}}}{{\rm{TS}}})}.$$

If the microRNA target gene of the functional annotation satisfied this condition, we defined the result as distinct, significant expression. In the formula, TB is the total number of mRNAs with functional annotations or path annotations, TS is the number of mRNAs corresponding to the differential expression of miRNA in the TB, B is the number of mRNAs annotated for a particular single function or a particular path, and S is the number of mRNAs annotated for a specific single function or a specific miRNA in a particular path. According to the first 8 GO functions and KEGG pathways with the smallest P values, we sorted the results and performed an enrichment analysis.

Among KEGG pathways, the pathway that is most closely related to hair follicle development is the Wnt signalling pathway. We separated microRNAs from their target genes in the Wnt pathway using the biogrid database and downloaded the network interaction, microRNAs, target genes, Wnt genes and signalling pathway interactions, which were distinguished by various colours, characteristics and attributes such as size, using Cytoscape software to obtain the functions in the Wnt pathway network analysis chart.

#### Analysis of main genes and the expression of microRNAs acting on the chorionic cycle

Real-time fluorescent quantitative PCR was performed using the FAST SYBR Green Master Mix Kit (Applied Biosystems, USA). Fluorescent quantitative primers were designed with primer5 (Table 4). PCR reactions were performed using an Agilent 3000XP Real Time PCR amplification instrument; the PCR reaction program was 95 °C for 20 sec, 1 cycle; 95 °C for 5 sec; 60 °C for 30 sec, 40 cycles; 95 °C for 1 min, 55 °C for 30 sec; and 95 °C for 30 sec, 1 cycle. Each sample was tested 3 times, and 3 blank controls were used for each primer. We used the 2^−ΔΔCt^ method for relative quantitation between samples, and the reference gene was β-actin. Using the statistical analysis software SPSS 17.0, we tested differences in the relative expression of keratin-associated protein genes in Arbas cashmere goat skin in different months using two methods: LSD and Duncan’s test. The results were expressed as the average value ± standard deviation.

**Table 4:** Primers used for PCR validation.

Gene name	Primer sequence	Product size
β-actin	Forward: GGCAGGTCATCACCATCGG	158 bp
	Reverse: CGTGTTGGCGTAAGAGTCTTT	
*CHP1*	Forward: GCTCTTTGGCTGGATGTGA	122 bp
	Reverse: GAGTGGTAGGTTGGGCAGAA	
*FZD6*	Forward: GGCAGACGAGAAACTGGAAC	126 bp
	Reverse: GTAAGCATCACCCACCACAC	
SIAH	Forward: GAGCCTTGCCATTTACAGGA	122 bp
	Reverse: TACGCCTCTTCTGGATGTGA	
*SMAD2*	Forward: TAAAAGTCCCAGGCATCACC	109 bp
	Reverse: ACCCCAGACAAGGAGCAGTA	
MiR-195	Forward: TGGTAGCAGCACAGAAATGTTGG	23 bp
